# Targeting Oncoproteins for Degradation by Small Molecule-Based Proteolysis-Targeting Chimeras (PROTACs) in Sex Hormone-Dependent Cancers

**DOI:** 10.3389/fendo.2022.839857

**Published:** 2022-03-18

**Authors:** Li Liu, Lihong Shi, Zhaodi Wang, Jun Zeng, Yue Wang, Hongtao Xiao, Yongxia Zhu

**Affiliations:** ^1^ Department of Clinical Pharmacy, Sichuan Cancer Hospital & Institute, Sichuan Cancer Center, School of Medicine, University of Electronic Science and Technology of China, Chengdu, China; ^2^ Department of Dermatology, The Affiliated Hospital of Southwest Medical University, Luzhou, China; ^3^ Department of Gynecology, People’s Hospital of Henan University, Henan Provincial People’s Hospital, Zhengzhou, China

**Keywords:** sex hormone-dependent cancers, PROTACs, small molecule inhibitors, estrogen receptors, androgen receptors

## Abstract

Sex hormone-dependent cancers, including breast, ovary, and prostate cancer, contribute to the high number of cancer-related deaths worldwide. Steroid hormones promote tumor occurrence, development, and metastasis by acting on receptors, such as estrogen receptors (ERs), androgen receptors (ARs), and estrogen-related receptors (ERRs). Therefore, endocrine therapy targeting ERs, ARs, and ERRs represents the potential and pivotal therapeutic strategy in sex hormone-dependent cancers. Proteolysis-targeting chimeras (PROTACs) are a novel strategy that can harness the potential of the endogenous ubiquitin-proteasome system (UPS) to target and degrade specific proteins, rather than simply inhibiting the activity of target proteins. Small molecule PROTACs degrade a variety of proteins in cells, mice, and humans and are an emerging approach for novel drug development. PROTACs targeting ARs, ERs, ERRs, and other proteins in sex hormone-dependent cancers have been reported and may overcome the problem of resistance to existing endocrine therapy and receptor antagonist treatments. This review briefly introduces the PROTAC strategy and summarizes the progress on the development of small molecule PROTACs targeting oncoproteins in sex hormone-dependent cancers, focusing on breast and prostate cancers.

## 1 Introduction

Great success has been achieved in drug discovery programs and human being health, particularly targeted therapy. There are two main types of targeted therapeutic drugs: monoclonal antibodies and small molecule drugs (1). Although targeted therapeutic drugs have fewer side effects than traditional chemotherapeutics, some limitations impede their widespread use (2). Monoclonal antibodies have potent specificity, however, their identification can be expensive, laborious, and time-consuming, meanwhile poor cell permeability limits their application (3). Whereas, treatment with small molecule drugs frequently results in the emergence of drug resistance (4, 5). New strategies to target protein degradation using small molecules have been developed (6, 7). One such attractive alternative technology involves PROteolysis TArgeting Chimeras (PROTACs), also known as protein degrader (7), which is an irreversible process of catalytic degradation of target proteins.

PROTACs can harness the endogenous ubiquitin-proteasome system (UPS) to target and degrade specific proteins (8, 9). PROTACs are heterobifunctional small molecules consisting of a target protein-binding ligand, an E3 recruiting ligand, and an interval linker. Rather than simply inhibiting the target protein activity, PROTACs can eliminate the entire target (9). The PROTAC strategy has been developed and validated for a range of targets, including kinases and protein targets that are “undruggable” *via* traditional inhibitors or non-enzymatic proteins (10, 11). Different studies have recently shown that embryonic ectoderm development (EED) -targeted or enhancer of zeste homolog 2-targeted (EZH2) -targeted PROTACs can potently degrade both target proteins and other core components of the polycomb repressive complex 2 (PRC2), suggesting that the PROTAC-mediated degradation mechanism can be a viable therapeutic modality (12–15). Due to this mechanism, the utilization of PROTACs can open new avenues for drug discovery efforts (11).

Over the last 20 years, PROTACs have evolved from peptides into small molecules that can degrade a variety of proteins in cells, mice, and humans (10, 11, 16). Small molecule PROTACs have gained the attention of both academic researchers and the pharmaceutical industry, including companies such as Arvinas, C4 Therapeutics, and Kymera Therapeutics (17). Some small molecule PROTACs have also undergone clinical trials (18, 19). In this review, we briefly introduce the PROTAC strategy and summarize the progress of small molecule PROTACs targeting oncoproteins in sex hormone-dependent cancers, with a particular focus on breast and prostate cancers. We also discuss the advantages and limitations of this emerging pharmacological modality.

## 2 Proteolysis Targeting Chimeras

**Figure 1:**
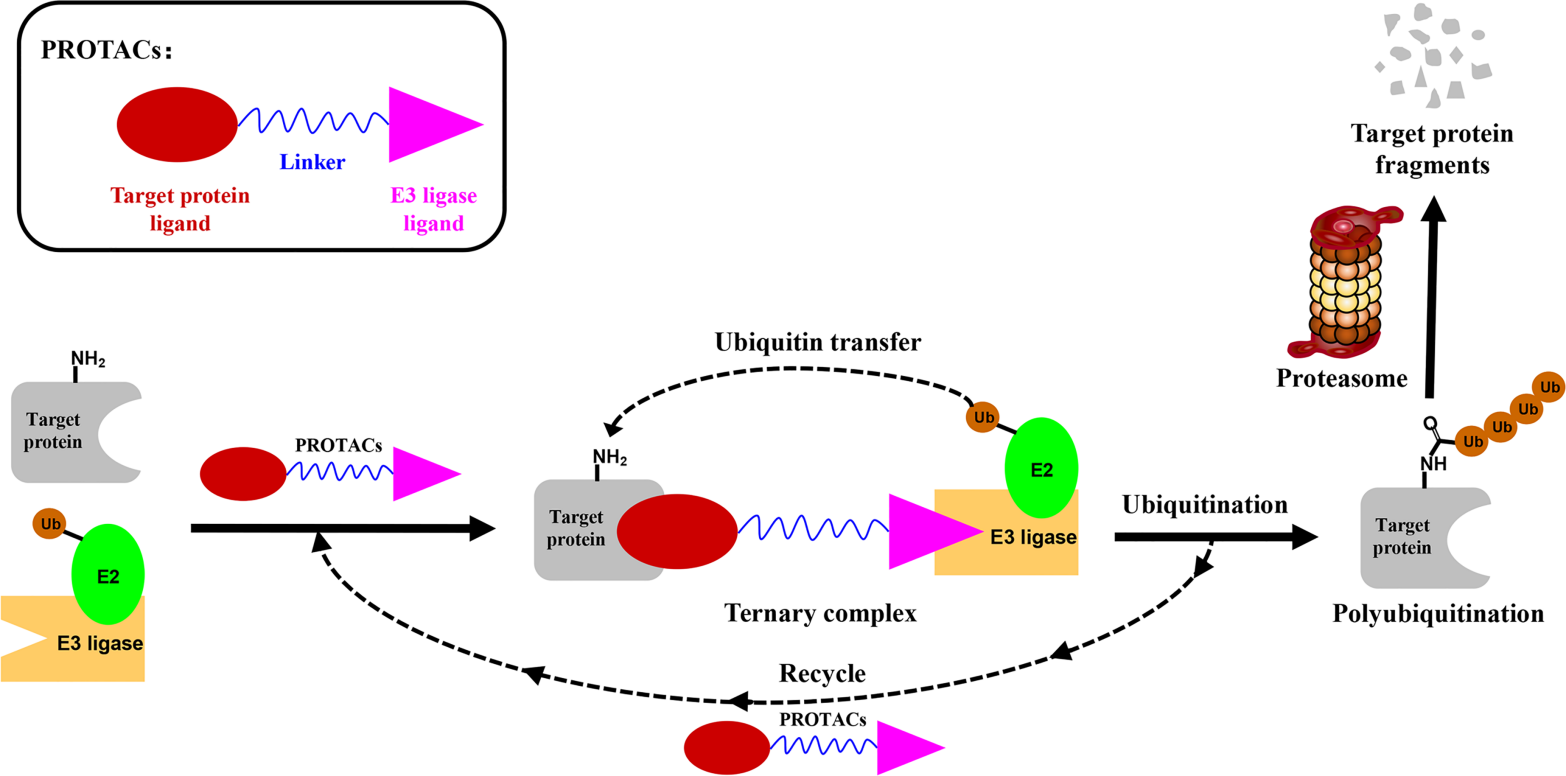
Schematic diagram of Proteolysis Targeting Chimeras (PROTACs) strategy. PROTACs comprise two ligands joined *via* a flexible chemical linker (blue font part), with one ligand (red font part) binding to the target protein and the other (violet font part) binding to the E3 ubiquitin ligase, followed by poly-ubiquitination and proteasome degradation of target protein.

PROTACs are bifunctional-hybrid molecules that comprise two ligands joined *via* a flexible chemical linker, with one of the ligands binding to the target protein and the other binding to the E3 ubiquitin ligase, facilitating poly-ubiquitination and subsequent proteasome-mediated degradation of the target protein (**Figure 1**). The concept of PROTACs was first proposed in cell lysates with peptide ligands by Deshaies and Crew in 2001 (9). Since then, PROTAC technology has evolved, and has been applied in cultured mammalian cells, *in vivo*, and even in clinical trials (10). There are more than 600 E3 ligase-encoding genes in the human genome. Numerous E3 ligases are employed in PROTAC technology, including cereblon (CRBN) (20), Von Hippel-Lindau (VHL) (21), a cellular inhibitor of apoptosis (cIAP) (22), and mouse double 2 homologue (MDM2) (23). CRBN and VHL are the most widely used because of the availability of drug-like small molecules that can recruit them (24–26). Other E3 ligases have also been studied and used to target protein degradation (27). In addition, diverse target proteins can be degraded efficiently by the utilization of PROTACs, including bromodomain and extra-terminal (BET) proteins (28), transcription factors (29), and tyrosine kinases [such as activin receptor-like kinases (ALK), Abelson tyrosine kinase (c-Abl), and Bruton’s tyrosine kinase (BTK)] (30).

### 2.1 First-Generation Peptide-Based PROTACs

**Figure 2:**
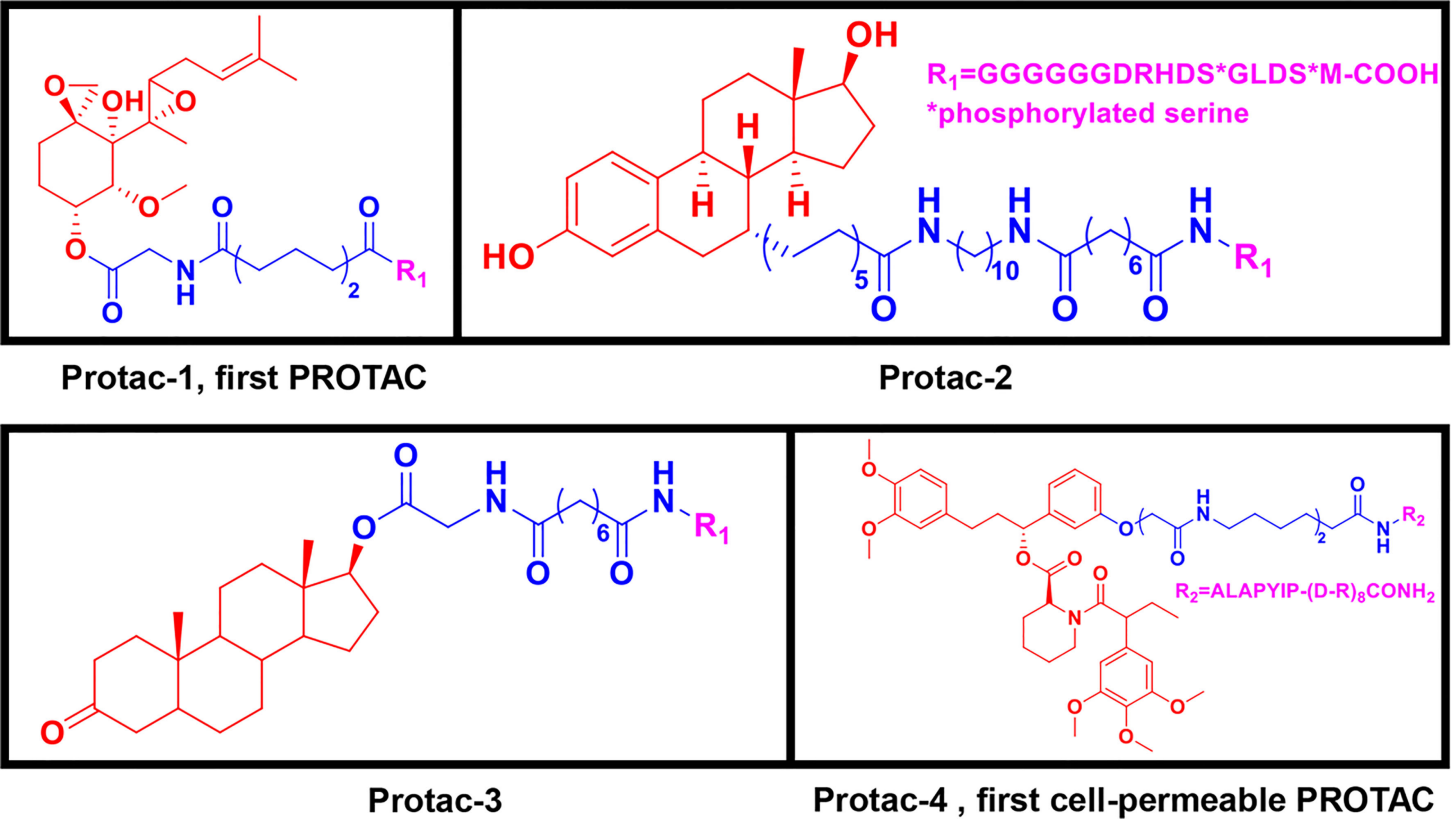
First-generation PROTACs, Peptide-based PROTACs. Chemical structures of Protac-1 (first PROTAC), Protc-2, Protac-3, and Protac-4 (first cell-permeable PROTAC).

Peptide-based PROTACs were the first-generation PROTACs, first introduced in 2001. They include Protac-1, which contains IκBα phosphopeptide as the E3 ubiquitin ligand recognized by the Skp1-Cullin-F (SCF) box complex (9). Protac-1 (**Figure 2**) recruits methionine aminopeptidase-2 (MetAP-2) to the SCF ubiquitin ligase, promoting MetAP-2 ubiquitination and inducing its degradation (9). In 2003, the PROTAC approach was used to target steroid hormone receptor proteins, including estrogen receptors (ERs) and androgen receptors (ARs) (31). Protac-2 and Protac-3 (**Figure 2**) consist of IκBα phosphopeptide and either estradiol or dihydroxytestosterone (DHT), which recruit ER or AR to the SCF ubiquitin ligase for ubiquitination and degradation, respectively (31). These three PROTAC molecules are all peptide-based and have cell permeability issues (9, 31).

To increase their cell permeability, Schneekloth et al. (32) designed the first cell-permeable peptide-based PROTAC (Protac-4) (**Figure 2**). Protac-4 contains ALAPYIP peptide with a ploy-D-arginine tag as the E3 ligase ligand, which is recognized by VHL. Protac-4 recruits the target protein, FK506-binding protein 12 (FKBP12), and induces its ubiquitination and subsequent degradation (32). Zhang et al. (33, 34) also developed a PROTAC molecule with a similar hydroxyl-proline peptide to hijack the VHL to target the ERα protein. This PROTAC can enter cells and inhibit breast cancer cell proliferation by degrading the ERα protein. Although additional strategies have been applied to peptide-based PROTAC molecules to improve cell permeability and new peptide-based PROTACs have been discovered, peptide-based PROTACs are still unattractive as drugs in clinical therapy owing to their vulnerable peptide bonds and poor delivery abilities (32, 35–37).

### 2.2 Second-Generation Small Molecule-Based PROTACs

**Figure 3:**
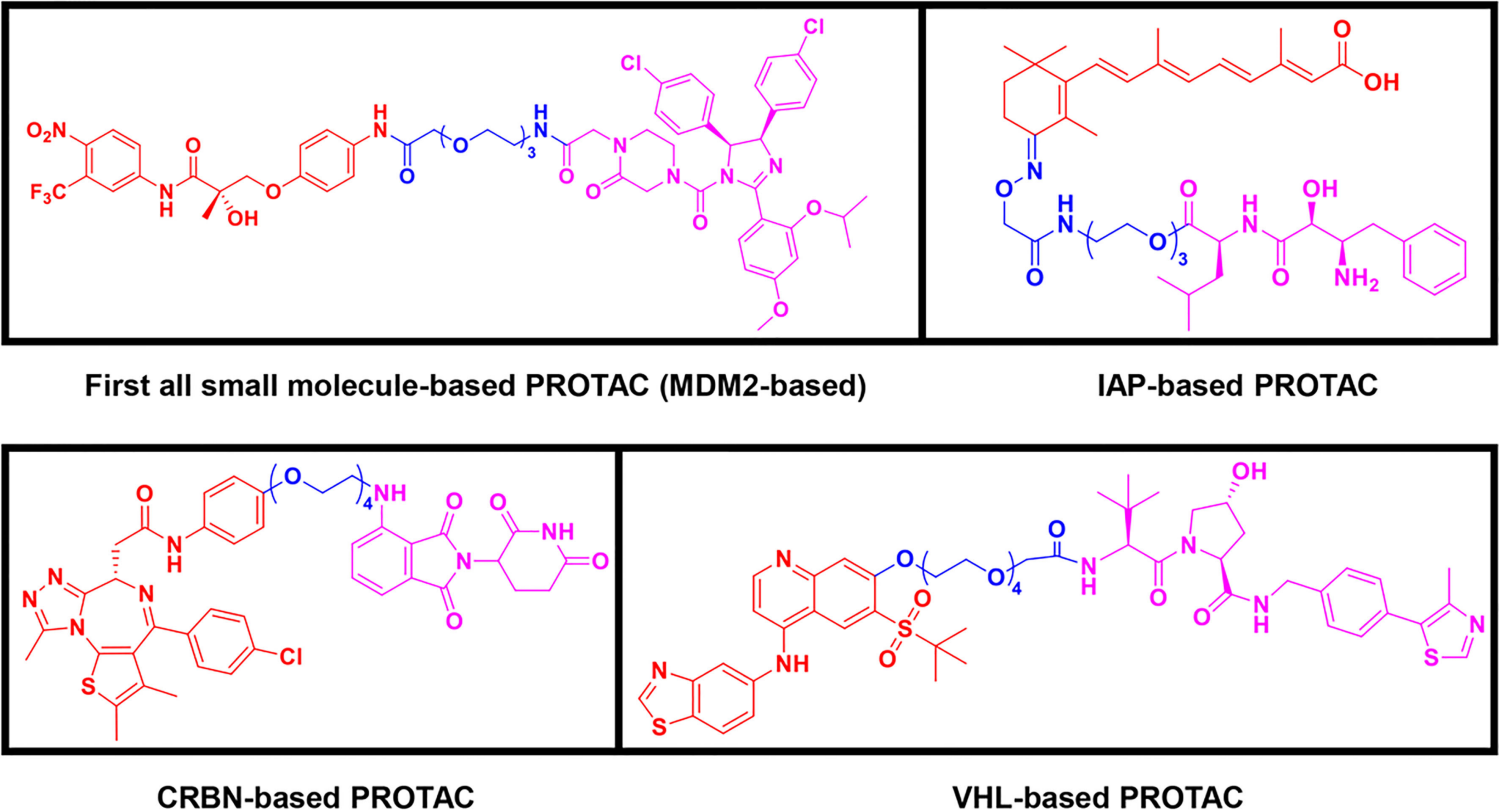
Second-generation PROTACs, Small molecule-based PROTACs. Chemical structures of first all small molecule-based PROTAC (MDM2-based), representative IAP-based PROTAC, representative CRBN-based PROTAC, and representative VHL-based PROTAC.

Following the early pioneering work, numerous small molecule-based PROTACs have been developed to overcome poor cell permeability and stability. In 2008, Schneekloth et al. (23) developed the first all small-molecule-based PROTAC targeting AR in HeLa cells. This PROTAC molecule (**Figure 3**) consists of a selective AR modulator (SARM) hydroxyflutamide, a non-steroidal AR ligand, and nutlin-3A, known as the MDM2 ligand, connected by a short soluble PEG linker. With an acceptable cell permeability, the SARM-nutlin PROTAC induced intracellular AR protein degradation in a UPS-dependent manner (23). All small-molecule-based PROTACs can efficiently induce intracellular protein degradation while bypass issues associated with peptide-based PROTACs.

Since then, small molecules have been widely used as E3 ligase ligands in PROTACs, including nutlin-3A (23) and Idasanutlin (38) as MDM2 ligand, VH032 (39), VHL ligand 8 (40), VH298 (41, 42) and their derivatives (43, 44) as VHL ligands, thalidomide (45), lenalidomide (46, 47), pomalidomide (45), and TD-106 derivatives (48) as CRBN ligands, and bestatin-methyl ester (ME-BS) (49), MV1 (50), and LCL161 (51) as IAP ligands. Several small molecule-based PROTACs have been reported based on these afore-mentioned small molecule ligands for E3 ligases, including PROTACs against BET proteins (28, 38), kinases (52–55), nuclear hormone receptors (17, 40, 43, 56–58), as well as additional proteins (59, 60).

Homo-PROTACs are a special type of small molecule-based PTOTACs, which dimerize one particular E3 ubiquitin ligase and then induce auto-degradation. The first homo-PROTAC, the representative compound CM11, was reported in 2017 (25). CM11 composed two identical VHL ligands, and induced proteasome-dependent self-degradation of VHL in different cell lines. Pomalidomide-based homo-PROTACs exhibit highly potent degradation of CRBN, with only minimal effects on Ikaros (IKZF1) and Aiolos (IKZF3) (61, 62). Homo-PROTACs provide a useful chemical tool to investigate the biological functions of different E3 ligases.

### 2.3 Third-Generation Controllable PROTACs

**Figure 4:**
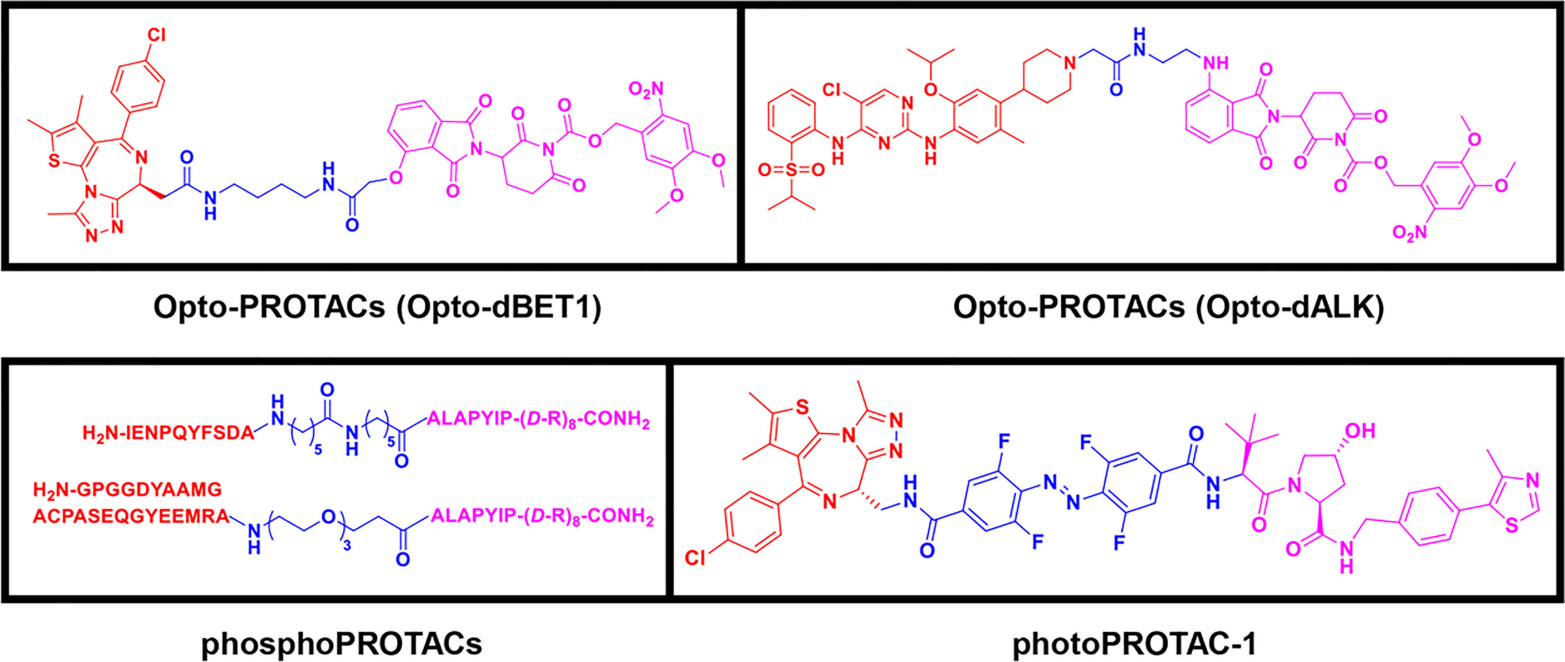
Third-generation PROTACs, Controllable PROTACs. Chemical structures of representative opto-PROTACs (opto-dBET1 and opto-dALK), representative phosphoPROTACs, representative photoPROTACs.

To overcome the off-tissue issues, which represent one of the major limitations of PROTACs, a new generation of controllable PROTACs was devised (63). Phospho-dependent PROTACs (phosphoPROTACs), the first controllable PROTACs, were developed by Hines et al. (64) in 2013 to specifically degrade targets by activating kinase-signaling (**Figure 4**). Since then, other research groups have independently reported light-inducible opto-PROTACs (**Figure 4**) and photo-switchable PROTACs (photoPROTACs, **Figure 4**), which use light, particularly ultraviolet A (UVA) or near-infrared, to achieve PROTACs control (65–68). These controllable PROTACs can potentially be used in clinical settings. However, their application is restricted to specific types of cancers because there is a lack of clear boundaries between tumor and normal tissues, and UV light can cause DNA damage and can penetrate tissues (8, 66).

## 3 Overview of Small-Molecule PROTACs in Sex Hormone-Dependent Cancers

Sex hormone-dependent cancers, including breast, ovarian, and prostate cancers, currently contribute to a high number of cancer-related deaths worldwide (69). Sex hormone-dependent cancers affect both females and males since sex steroid hormones share common features (70). Some hormones can activate relevant pathways by binding to related receptors, including ER and AR, and then promote tumor occurrence, development, and metastasis (71). Therefore, ER and AR are potential therapeutic targets in sex hormone-dependent cancers (72).

### 3.1 Small-Molecule PROTACs Targeting AR in Prostate Cancer

**Figure 5:**
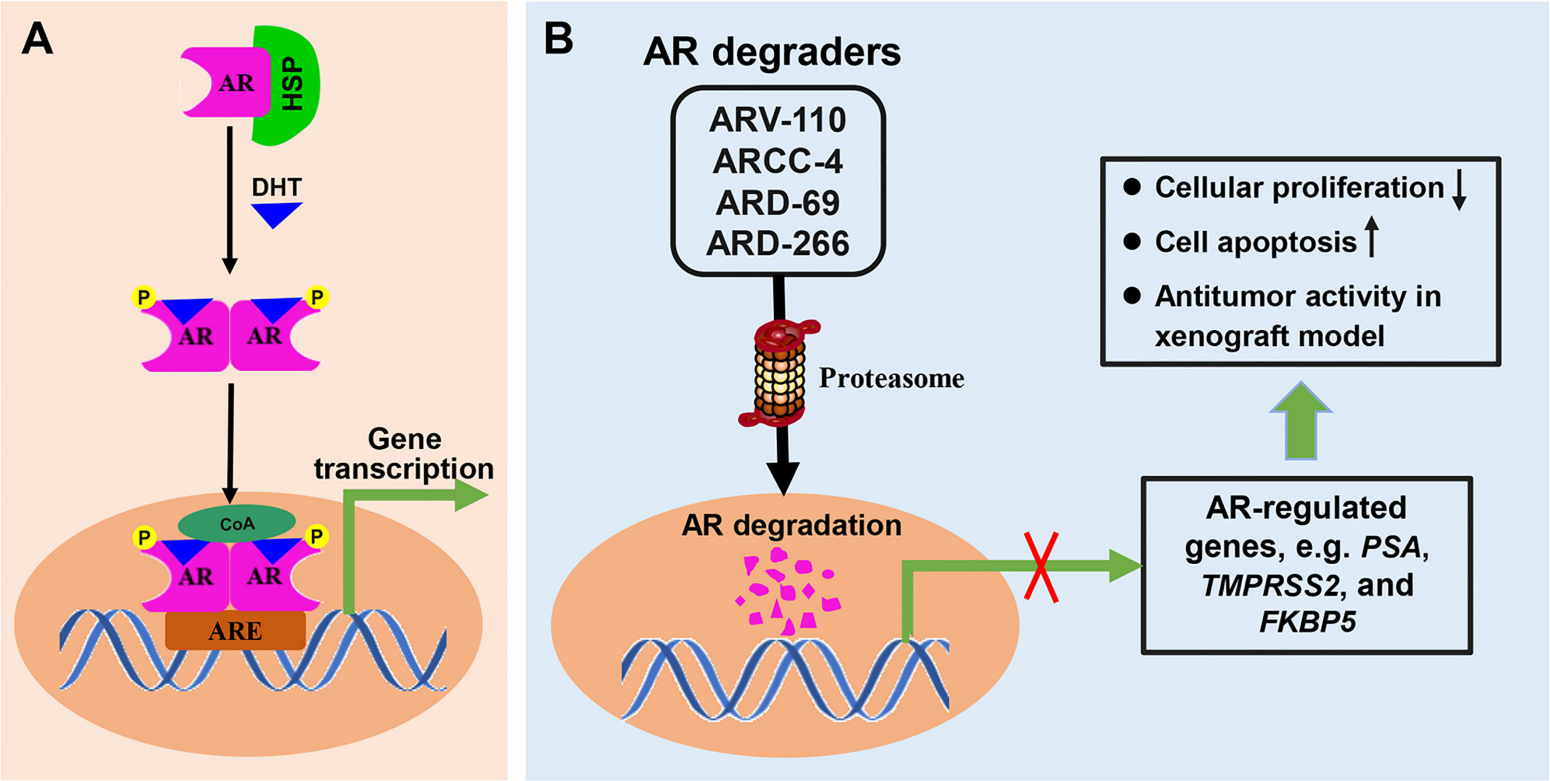
The mechanisms involved in AR signal pathway and PROTACs targeting AR. **(A)** Dihydrotestosterone (DHT) binds to inactivated AR, and cause the dissociation of heat shock protein (HSP) from AR-HSP complex. DHT-bound AR translocate to the nucleus after suffer phosphorylation and dimerization, then interacts with the androgen response element (ARE) and control target gene transcription. **(B)** PROTAC AR degraders can induce proteasome-mediated degradation of AR protein, and effectively suppress the mRNA levels of AR-regulated genes, which resulted in cellular proliferation inhibition, induction of cell apoptosis, and antitumor activity in xenograft model.

Prostate cancer (PC) is the second leading cause of cancer-associated mortality in males in developed countries after lung cancer, and the incidence rate is increasing in developing countries (73, 74). Dihydrotestosterone (DHT) binds to inactivated AR, and cause the dissociation of heat shock protein (HSP) from AR-HSP complex. DHT-bound AR then translocate to the nucleus after suffer phosphorylation and dimerization. As a nuclear transcription factor, AR binds to the androgen response element (ARE) in DNA and starts the transcription of target genes after recruiting transcription regulators (**Figure 5A**). AR is the main driving force in PC development and has been identified as a pivotal therapeutic target. Endocrine therapy is one of the important strategies for PC treatment, including abiraterone by blocking androgen synthesis and AR antagonist enzalutamide by inhibiting AR function. However, most patients with PC will inevitably progress to castration-resistant PC (CRPC) (75). For metastatic CRPC, the main treatment plan involves using the small molecule chemotherapy drugs docetaxel and carbataxel (75), combined with the androgen small molecule inhibitors abiraterone and enzalutamide (76).

Unfortunately, approximately 25% of CRPC patients do not respond to these treatments, and the vast majority of responsive patients will ultimately develop resistance. AR signal pathway can be activated in CRPC because of AR mutation, AR amplification and AR alternate splicing variants formation, which cause the failure of traditional endocrine therapy. Nonetheless, small-molecule PROTACs targeting AR, which may overcome the problem of resistance to existing drug treatments, have been recently reported and provide an attractive direction (77). A number of other small-molecule PROTACs targeting AR have been discovered since the discovery of the first in 2008 (23). PROTAC AR degraders can induce proteasome-mediated degradation of AR protein in prostate cancer, block AR signaling and suppress the mRNA levels of AR-regulated genes, which results in cellular proliferation inhibition, induction of cell apoptosis, and antitumor activity in a xenograft model (**Figure 5B**). The subsequent sections of this review introduce these representative degraders.

#### 3.1.1 ARV-110

ARV-110 (**Table 1**) is a small-molecule PROTAC drug developed by Arvinas Inc. (New Haven, USA). ARV-110 uses PROTAC technology to degrade AR protein and has been developed as a potential treatment for metastatic CRPC, which is the second most prevalent cancer in men (78). AR activates the transcription of a variety of proteins and is closely associated with cell proliferation and apoptosis following binding to its ligand, thus fueling tumor progression. ARV-110 completely degraded AR in prostate vertebral body cancer (VCaP) and LNCaP cell lines, giving a half degradation concentration (DC_50_) of < 1 nM (77). ARV-110 inhibited the expression of prostate-specific antigen (*PSA*) and FK506 binding protein 5 (*FKBP5*), inhibited AR-dependent cell proliferation, and induced apoptosis in VCap cells. It also showed plasma PSA reduction potency similar to that of the traditional AR antagonist, enzalutamide, but at lower doses, and demonstrated efficacy in enzalutamide-resistant PC xenograft models (78). As an orally bioavailable AR PROTAC degrader, ARV-110 was evaluated for safety, tolerance, and pharmacokinetics in a phase I clinical trial (NCT03888612) in early 2019, and the results highlighted its acceptable safety and antineoplastic activity in a heavily pretreated cohort with metastatic CRPC (18, 79). A phase II clinical trial (NCT03888612) is currently underway to estimate the therapeutic potential of ARV-110 in men with metastatic CRPC who have failed to standard treatment.

**Table 1:** Components and properties of small-molecule PROTACs in sex hormone-dependent cancer.

Target/Compound	Chemical structure	Target ligand	E3 ligase	E3 ligand	Degradation in cells		Refs
					DC_50_	D_max_	
**AR**							
ARV-110	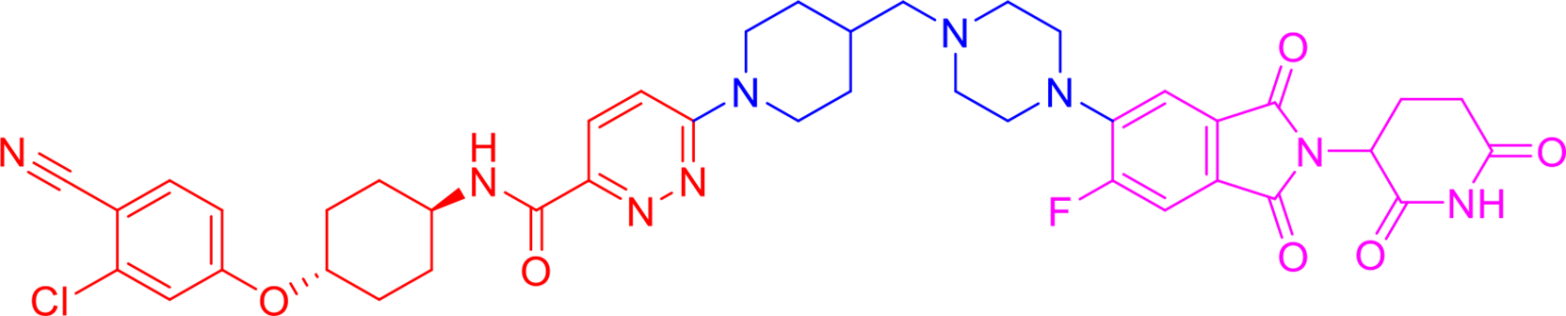	AR antagonist	CRBN	Thalidomide	<1 nM in LNCaP and VCaP cells	–	(77–79)
ARCC-4	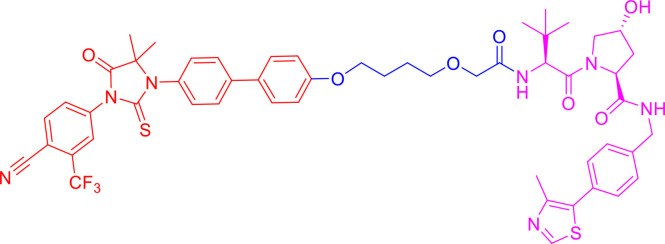	Enzalutamide	VHL	VHL032	~ 5 nM in LNCaP and VCaP cells	> 98%	(80)
ARD-69	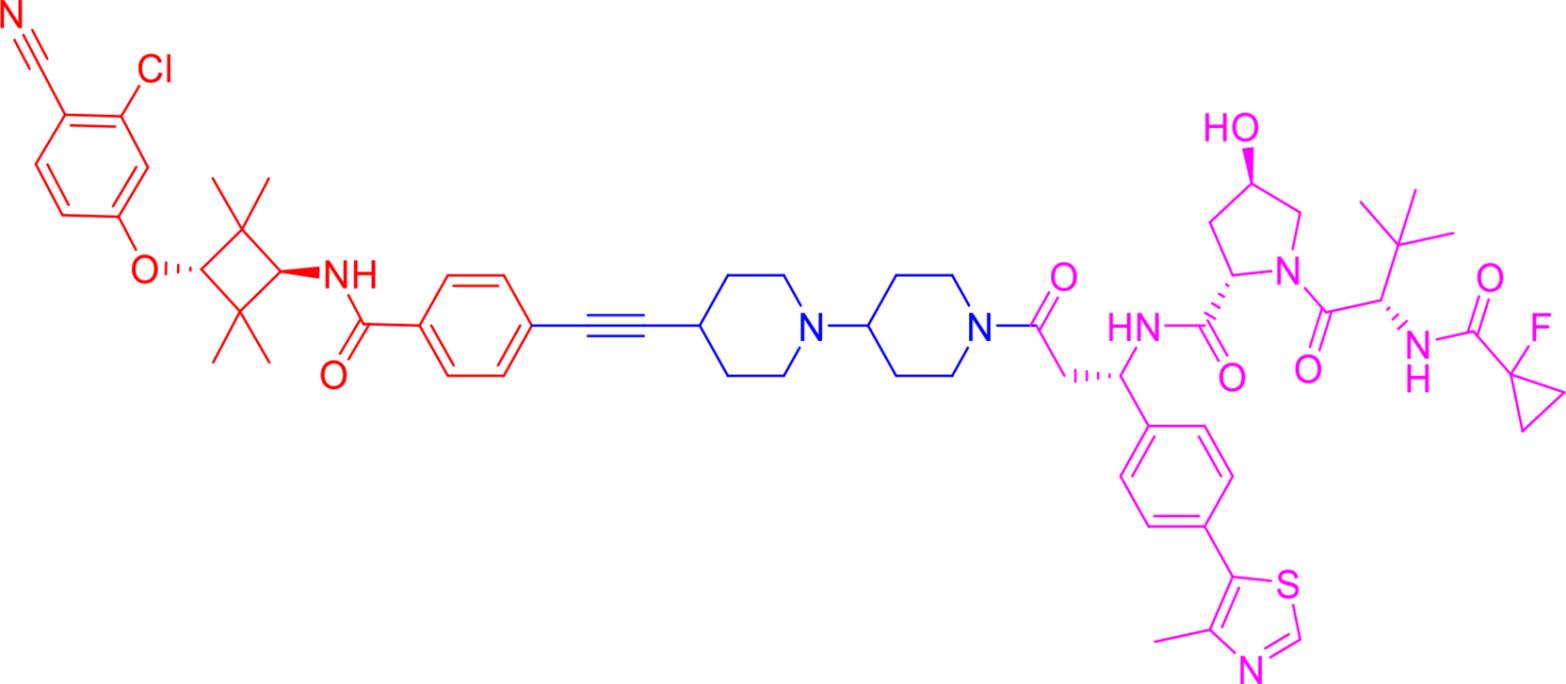	AR antagonist	VHL	VHL-e	<1 nM in LNCaP and VCaP cells	> 95%	(56)
ARD-61	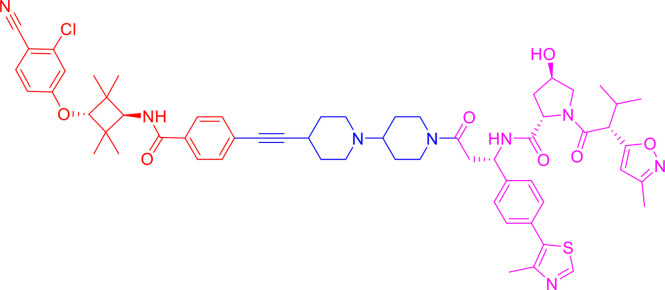	AR antagonist	VHL	VHL-d	7.2 nM in LNCaP and 1.0 nM in VCaP cells	> 95%	(56, 81)
ARD-266	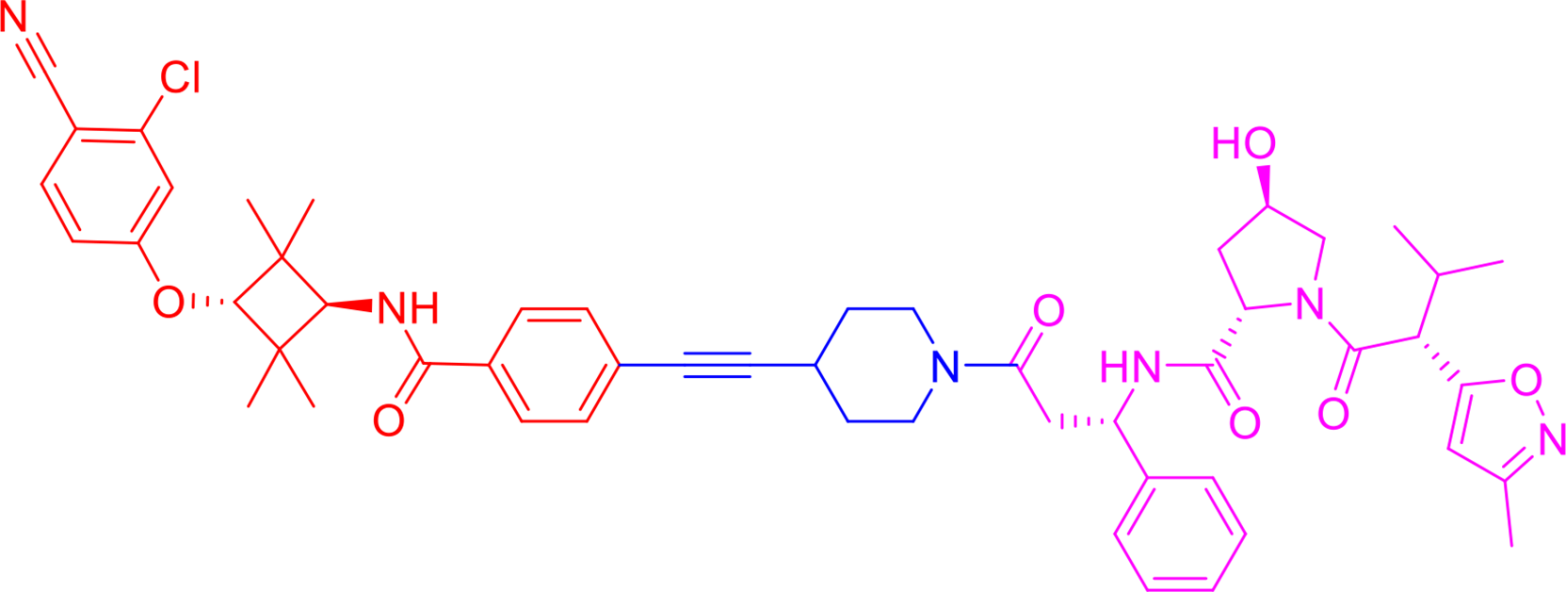	AR antagonist	VHL	VHL-g	0.5 nM in LNCaP and 1.0 nM in VCaP cells	> 95%	(40, 82)
**ERα**							
ARV-471	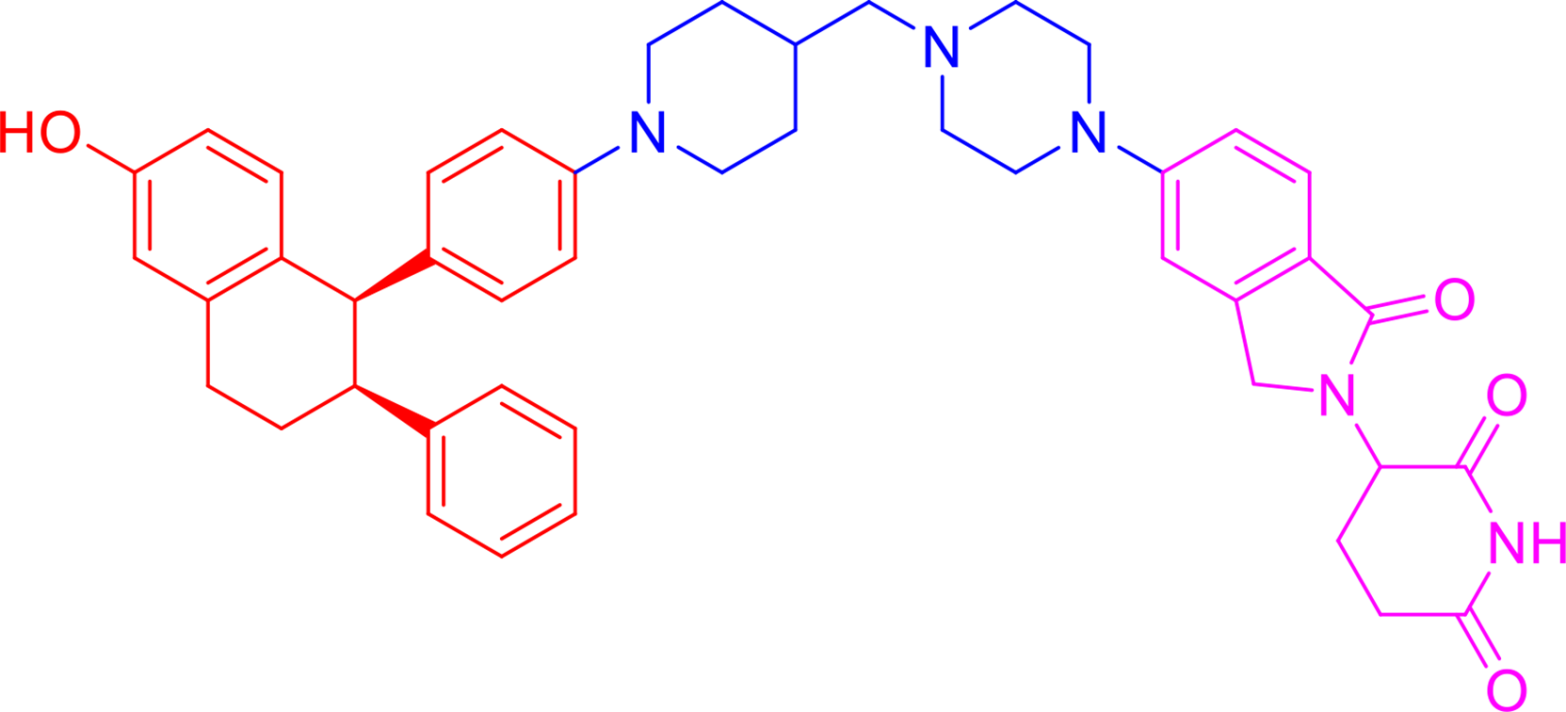	ER agonist	CRBN	Lenalidomide	1.8 nM in MCF-7 cells	–	(19, 83)
ERD-308	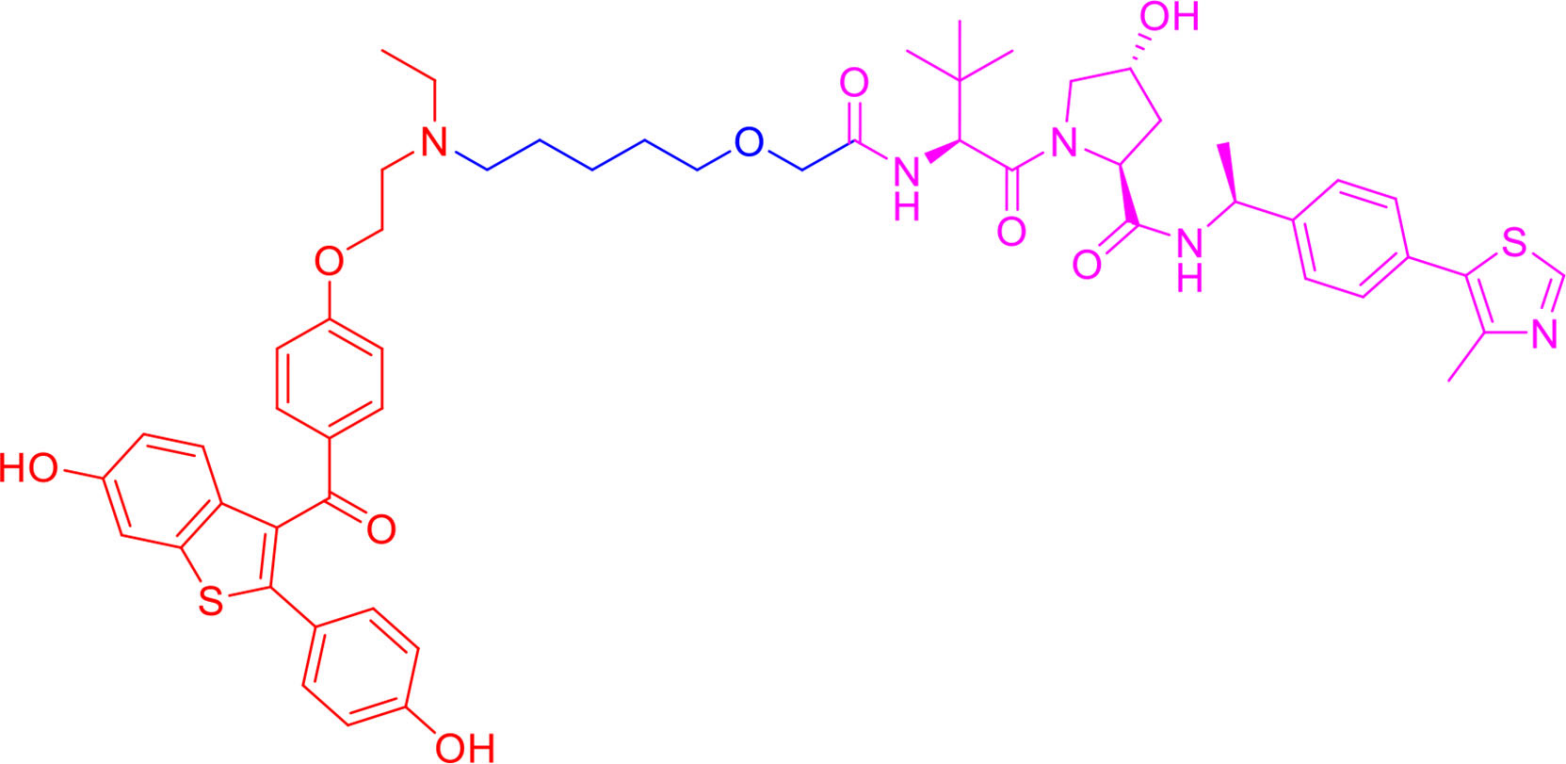	Raloxifene derivative	VHL	VHL032 derivative	0.17 nM in MCF-7 cells; 0.43 nM in T47D cells	> 95%	(57)
ERD-148	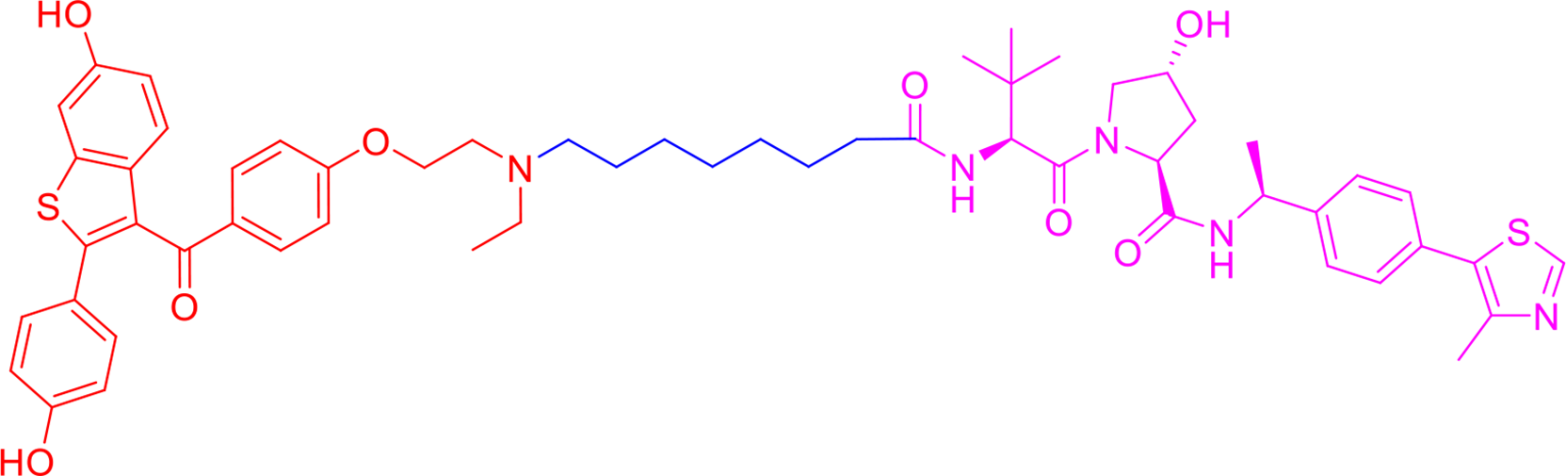	Raloxifene derivative	VHL	VHL032 derivative	<10 nM in MCF-7 WT and Y537S cells	–	(57, 84)
SNIPER(ER)-3	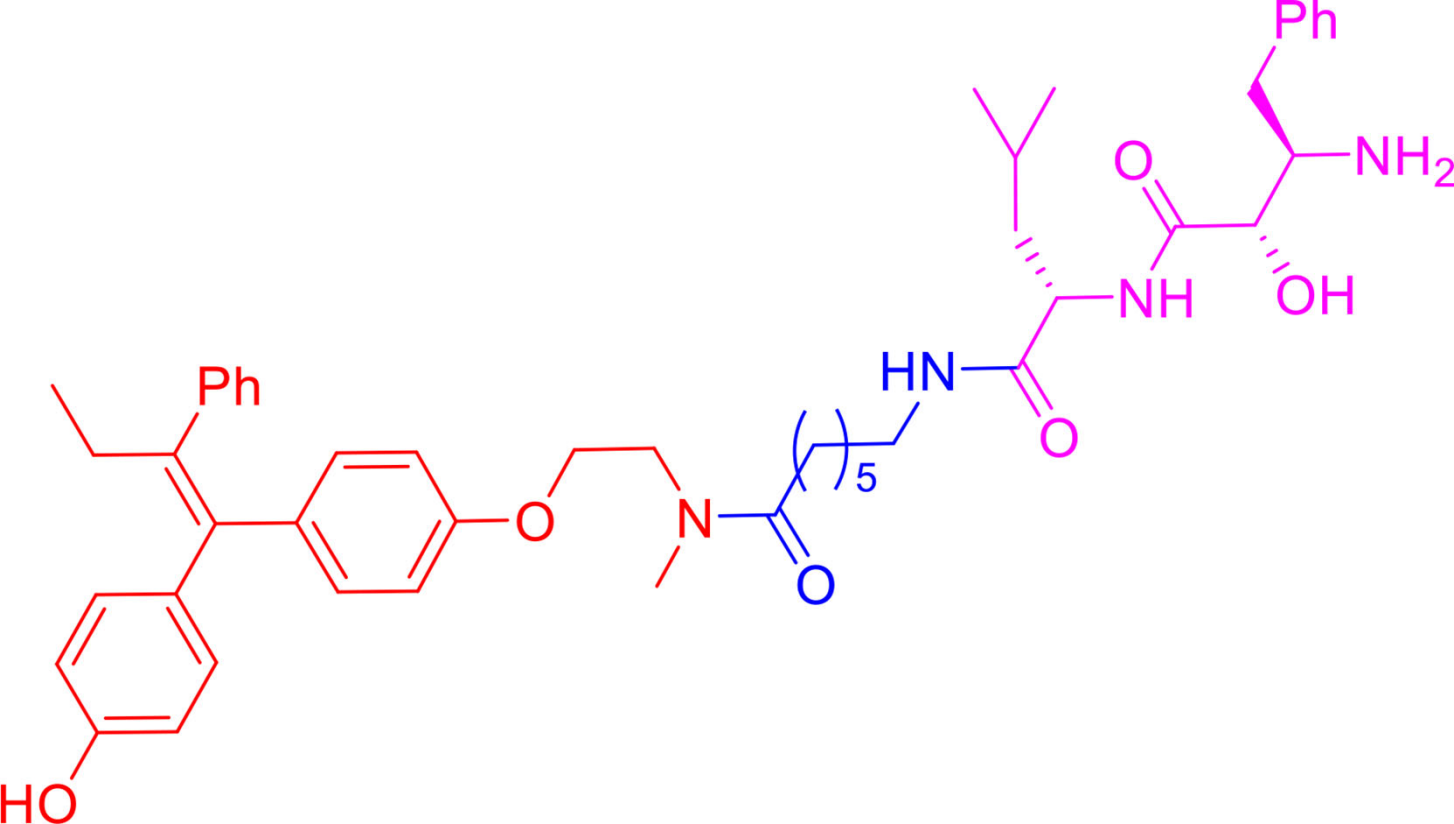	0-OHT	IAP	Bestatin	<10 µM in MCF-7 cells	–	(85, 86)
SNIPER(ER)-87	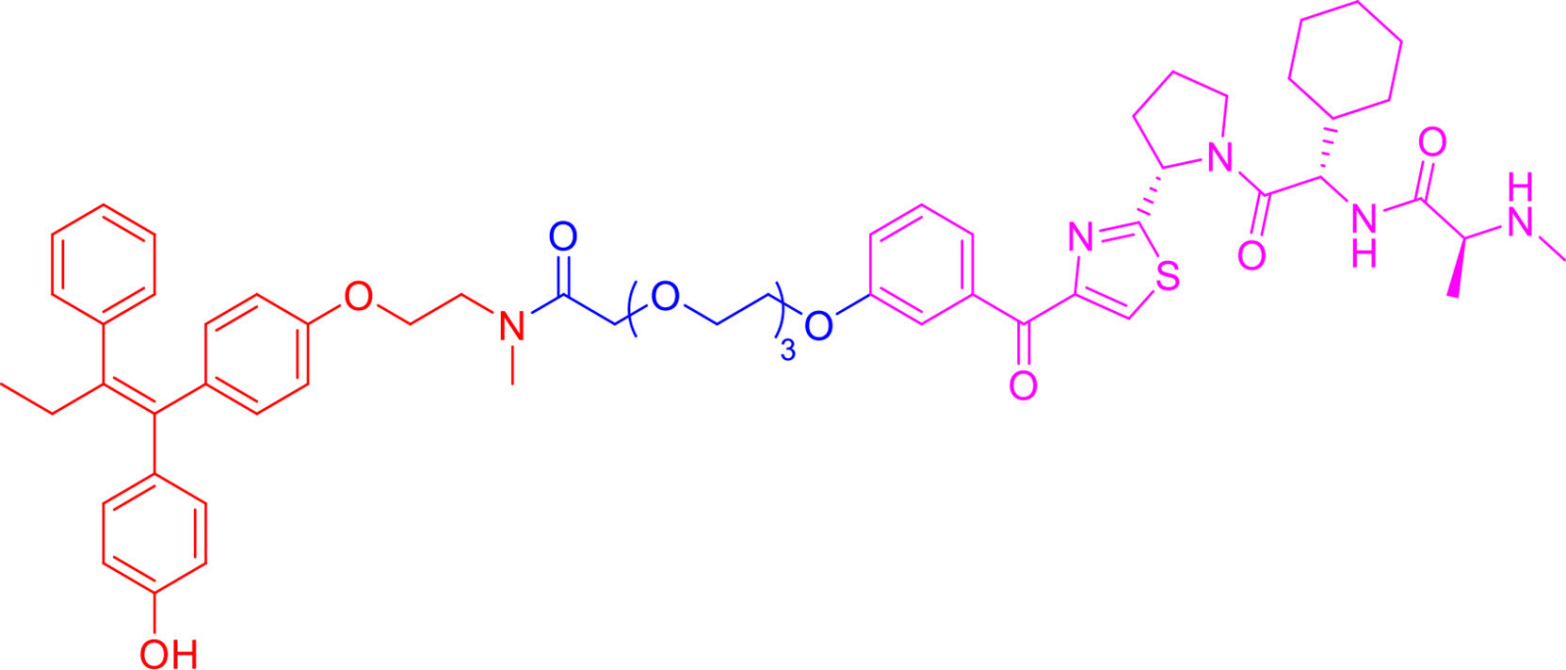	0-OHT	XIAP	LCL161 derivative	<3 nM in MCF-7 cells	–	(87)
PROTAC-like SERDs cpd. **17e**	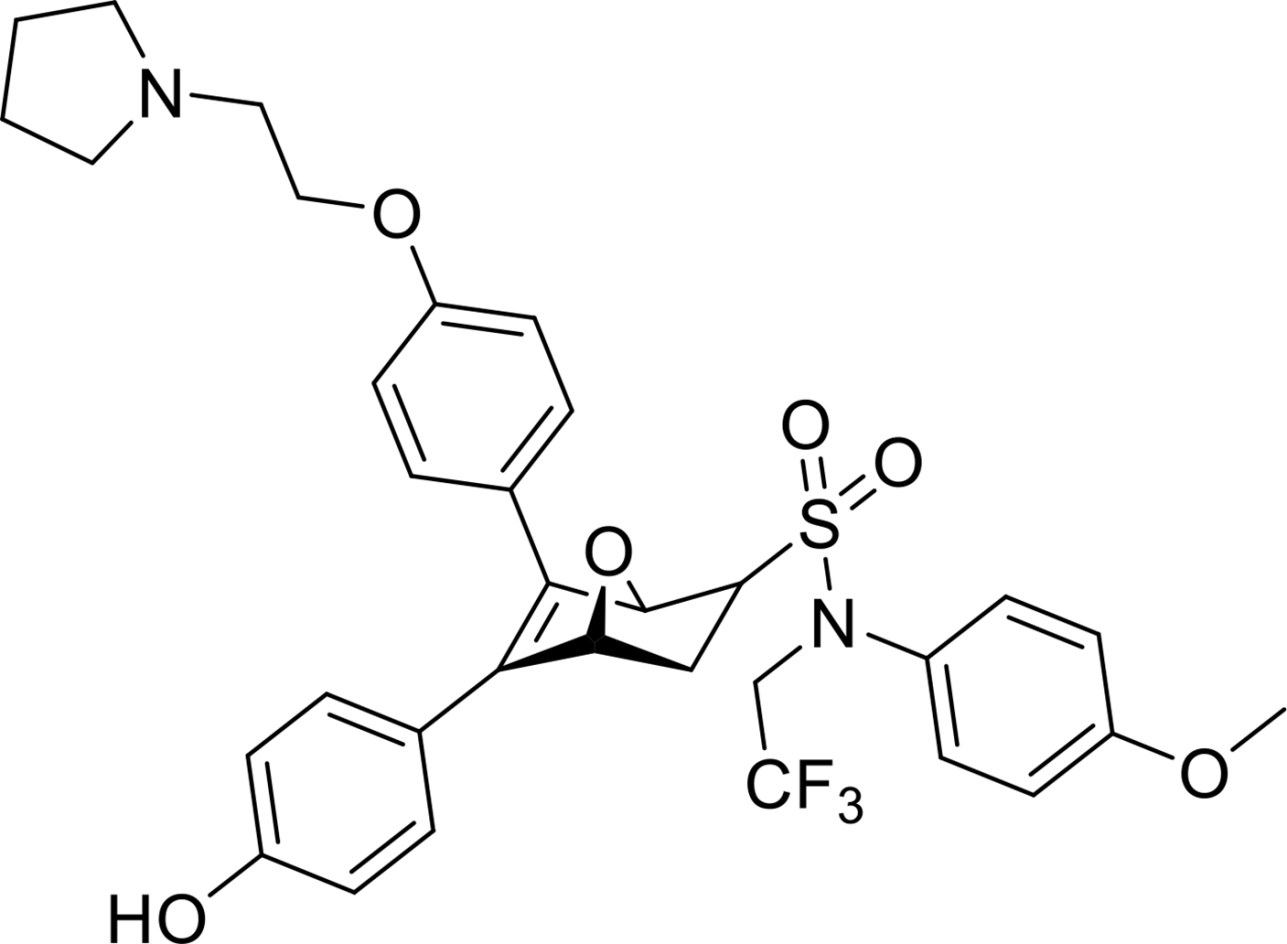	–	–	–	<0.5 µM in MCF-7 cells	> 95%	(88)
**ERRα**							
PROTAC cpd. **29**	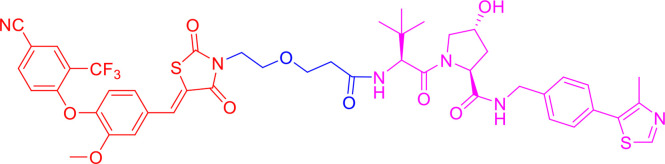	Thiazolidinedione-based ligand	VHL	VHL032	~100 nM in MCF-7 cells	86%	(43)
PROTAC cpd. **6c**	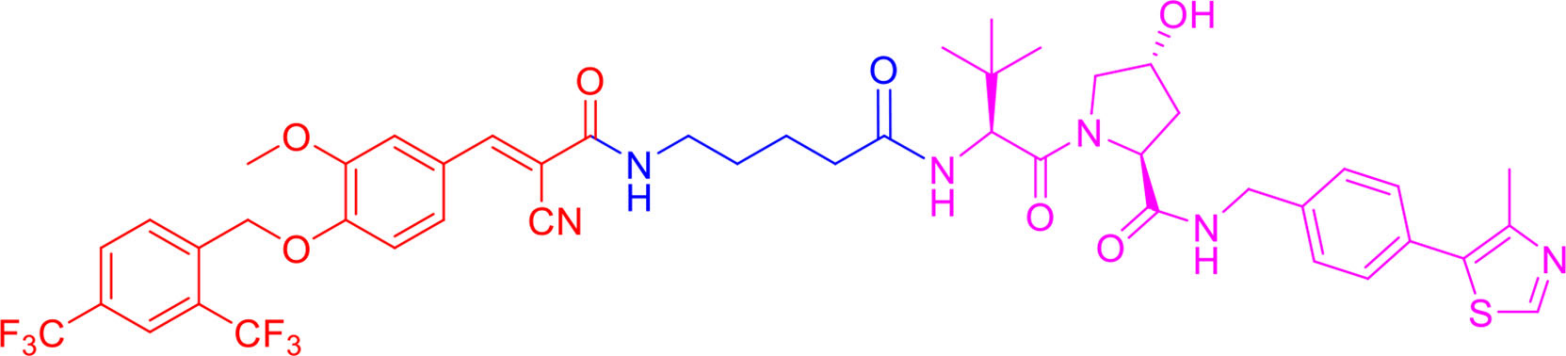	XCT790 derivative	VHL	VHL032	<3 nM in MDA-MB-231 cells	–	(89)
**Other proteins**							
ARV-771	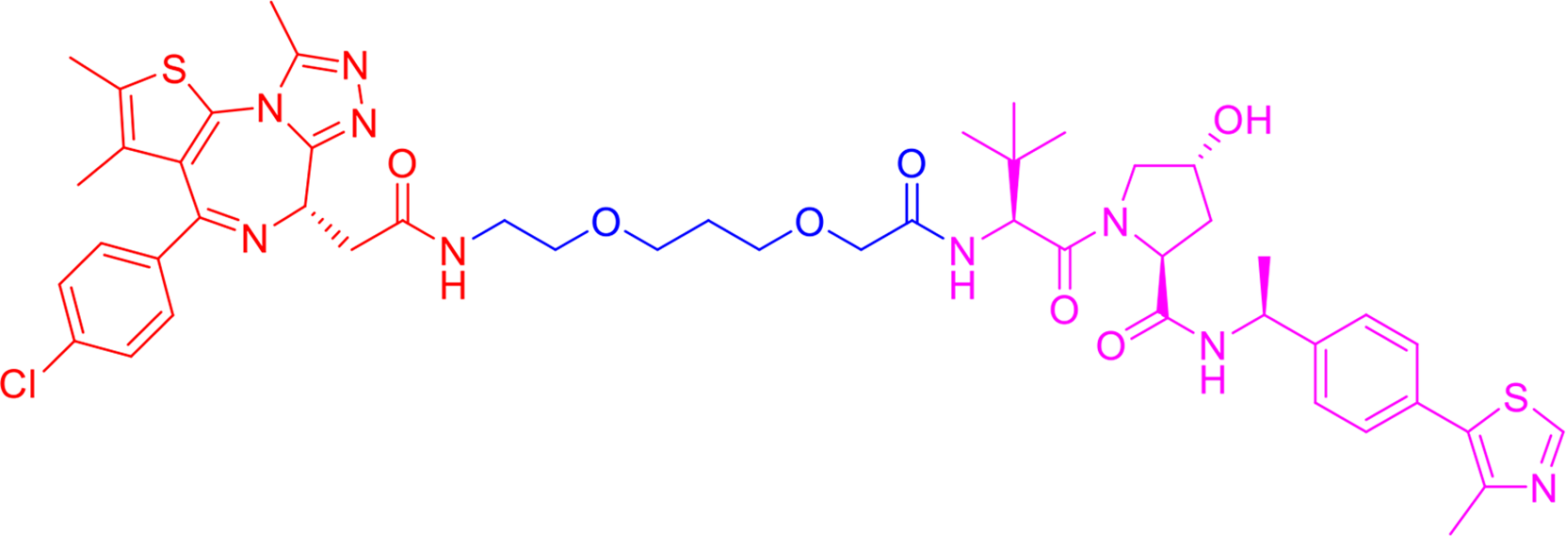	BET ligand	VHL	VHL032 derivative	<5 nM in 22Rv1, VCaP, LnCaP95 cells	–	(90)
PROTAC cpd. **3**	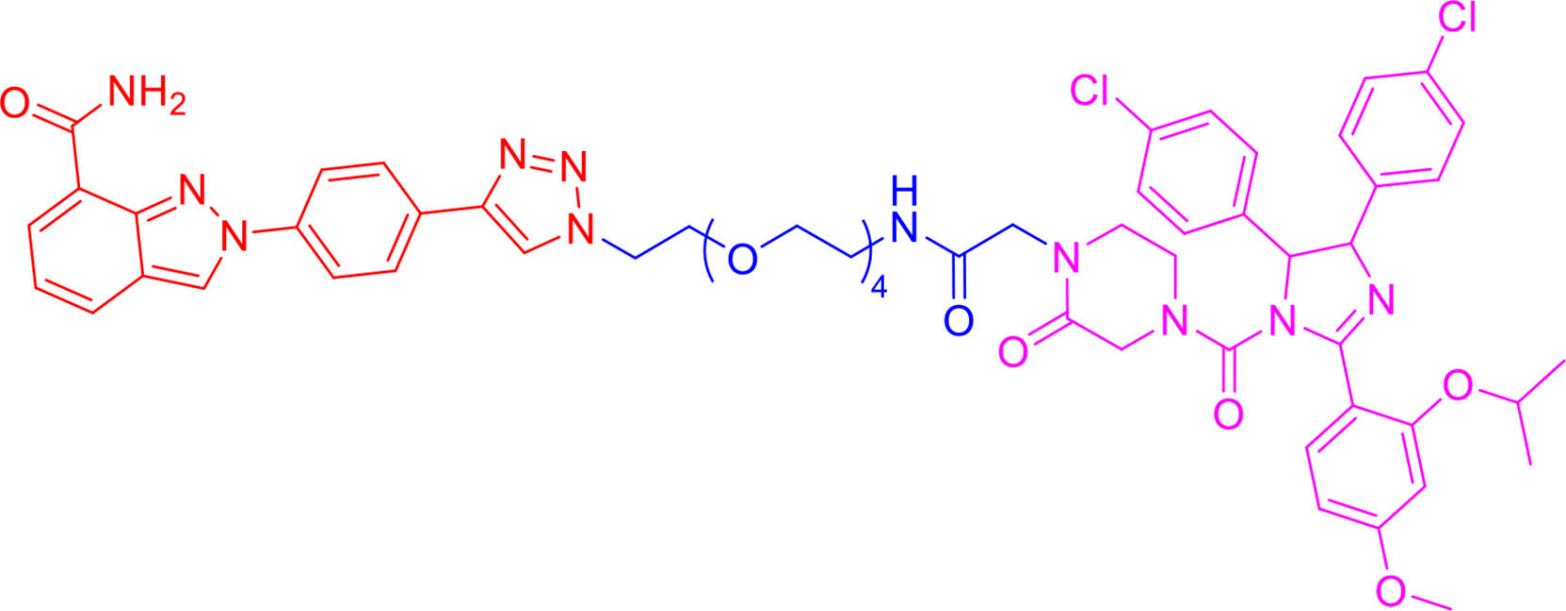	Niraparib (PARP1 binding moiety)	MDM2	Nutlin-3a derivatives	–	–	(91)

#### 3.1.2 ARCC-4

In 2018, Salami et al. (80) synthesized a variety of enzalutamide-based VHL-recruiting PROTACs targeting AR, and ARCC-4 (**Table 1**) was screened out using cellular models of drug resistance. ARCC-4 was identified as a highly efficient degrader, with DC_50_ of about 5 nM and a maximum degradation (D_max_) of more than 98%, even in cells expressing high levels of AR protein. After head-to-head comparisons between the currently approved AR antagonist enzalutamide and ARCC-4 in different cell models of PC resistance, the antitumor efficacy of ARCC-4, evaluated by *PSA* expression reduction, induction of cell apoptosis, and AR-dependent cell proliferation inhibition, outperformed enzalutamide. Interestingly, ARCC-4 effectively degraded clinically relevant ARs with different point mutants, including F876L, T877A, L702H, H874Y, and M896V, and remained active despite elevated androgen levels. ARCC-4 had better anti-proliferative effects in AR mutant cancers, whereas enzalutamide failed.

#### 3.1.3 ARD-69

Han et al. (56) designed, synthesized, and evaluated a series of PROTAC AR degraders using five different AR antagonists, ligands for high-affinity VHL E3 ligases, and an optimized linker, ARD-69 (**Table 1**) was identified as the most effective AR degrader. ARD-69 achieved DC_50_ values of 0.86, 0.76, and 10.4 nM in LNCaP, VCaP, and 22Rv1, respectively. After 24 h of treatment, ARD-69 almost completely degraded AR in LNCaP and VCaP cells, with the concentration reaching below 1 nM. ARD-69 also effectively and dose-dependently inhibited *PSA*, transmembrane protease serine 2 (*TMPRSS2*), and *FKBP5* expression. Furthermore, ARD-69 inhibited LNCaP, VCaP, and 22Rv1 cell proliferation, with efficiencies 100 times higher relative to enzalutamide. Additionally, ARD-69 effectively reduced AR and PSA protein expression in VCaP xenograft tumor tissues. Altogether, these data demonstrate that ARD-69 is an extremely potent AR degrader in treating metastatic CRPC.

#### 3.1.4 ARD-266

Wang et al. (40) also investigated how the binding affinity of the VHL ligand portion of the VHL protein influences the potency and efficiency of PROTAC degraders. Using the PROTAC degrader ARD-61 (**Table 1**) with a high-affinity VHL ligand (56, 81), they discovered another more potent PROTAC AR degrader, ARD-266 (**Table 1**), consisting of a low-affinity VHL ligand, a reoptimized shorter linker, and the same AR antagonist (40). ARD-266 effectively induced AR protein degradation in the AR-positive (AR+) PC cell lines LNCaP, VCaP, and 22Rv1, yielding DC_50_ values of 0.2–1 nM. In addition, AR protein levels in LNCaP and VCap cells decreased by more than 95% following treatment with 10 nM ARD-266 for 6 h. Notably, ARD-266 effectively inhibited AR-regulated gene expression in a dose-dependent manner. This was the first study to demonstrate that, even using a low-affinity ligand with nanomolar levels of E3 ligase complex, the PROTAC degrader can efficiently degrade the target protein. Development of additional AR degraders is currently underway (82).

### 3.2 Small-Molecule PROTACs Targeting ERα in Breast Cancer

**Figure 6:**
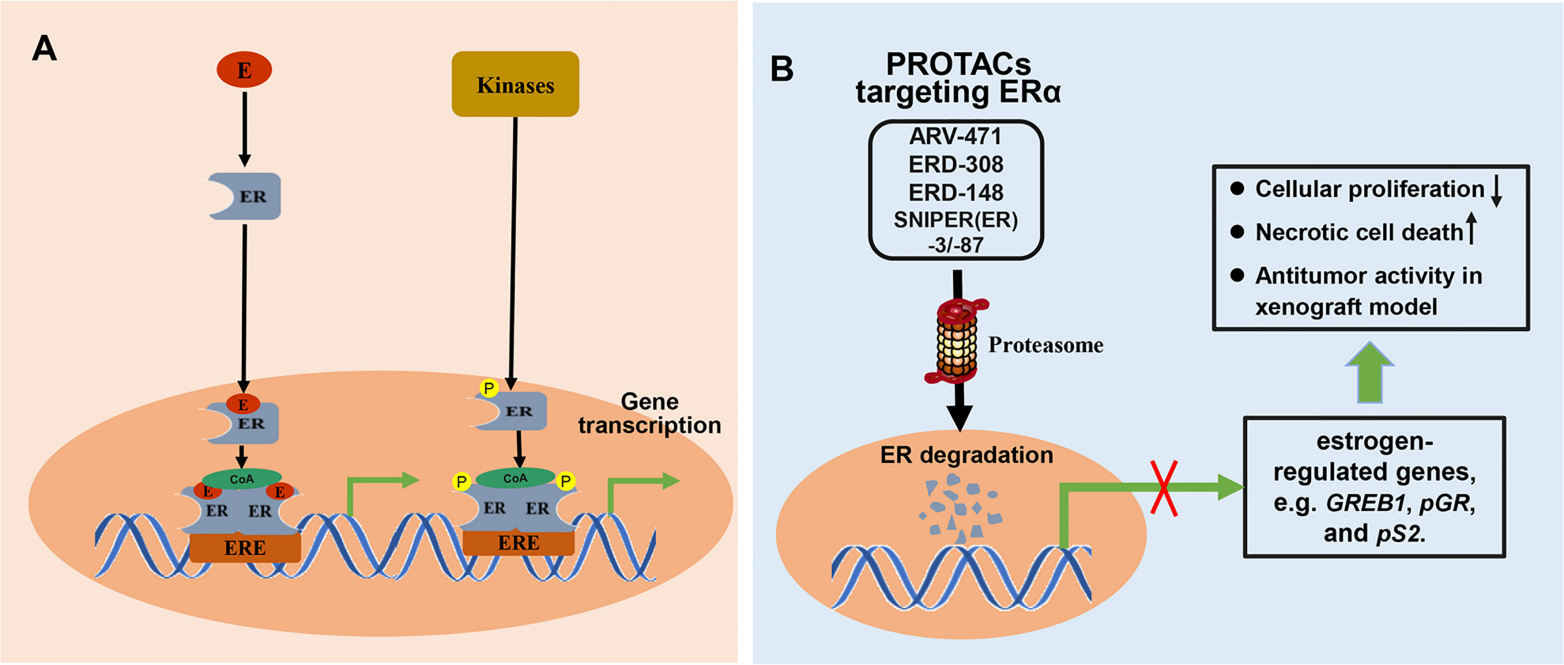
The mechanisms involved in ER signal pathway and PROTACs targeting ER. **(A)** Classically, ligands, like estrogen (E), bind to the ER to form ligand-bound ER. ligand-bound ER dimerizes and binds to estrogen response elements (ERE), and then activates gene transcription after recruiting coactivator (CoA). Besides, ER can be phosphorylated by related kinases and regulates gene transcription in a ligand independent pathway. **(B)** PROTAC targeting ER can induce proteasome-mediated degradation of ER protein, and effectively suppress the mRNA levels of estrogen-regulated genes, which resulted in cell proliferation inhibition, induction of necrotic cell death, and significant antitumor activity in xenograft model.

Breast cancer is one of the most common malignant tumors in women and is associated with the highest cancer-related mortality. Approximately 70% of breast cancers are ER-positive (ER+) (92). The ERs located in the nucleus, including ERα and ERβ, can directly bind to DNA or indirectly bind to DNA through corresponding transcription factors to regulate the transcription of target genes and exert biological effects. Besides, ER can be phosphorylated and regulate gene transcription in a ligand independent pathway (**Figure 6A**). ER is, therefore, an important therapeutic target for ER+ breast cancer. Although approved treatments were relatively successful, resistance to previous anti-estrogen therapy is developing in ER+ breast cancer. Fulvestrant, a selective estrogen receptor degrader (SERD), is the only ER-degrading agent approved for the treatment of ER+ breast cancer following anti-estrogen therapy. Though fulvestrant is beneficial for treating ER+ breast cancer, clinical trials show that it has poor solubility and cannot be orally administered. In clinical practice, fulvestrant can only be administered intramuscularly, limiting the total amount of drug intake and resulting in incomplete receptor blockade. To improve drug delivery, new ER-degrading agents are under continuous development. The recent use of PROTAC technology to degrade ER in breast cancer cells has attracted increasing attention. PROTAC targeting ERα can degrade both ERα proteins no matter whether ERα-encoding genes is mutated, and reduce the expression of estrogen-regulated genes, which resulted in cell proliferation inhibition, induction of necrotic cell death, and significant antitumor activity in MCF7 and patient-derived xenograft models (**Figure 6B**).

#### 3.2.1 ARV-471

ARV-471 (**Table 1**) is a small-molecule PROTAC degrader co-developed by Arvinas Inc. and Pfizer to target ER (19). It is used for locally advanced or metastatic ER+/human epidermal growth factor receptor 2 (HER2) negative breast cancer, and is currently in phase II clinical trial (NCT04072952). ARV-471 degraded ER in ER+ breast cancer cell lines, with a DC_50_ of 1.8 nM (83). ARV-471-mediated ER degradation reduced the expression of estrogen-regulated genes and inhibited the proliferation of estrogen-dependent cell lines, including MCF7 and T47D cells. In addition, ARV-471 degraded clinically related *ESR1* mutants (Y537S and D538G) and inhibited the growth of cell lines with these mutations. It also showed significant antitumor activity in an estrogen-dependent MCF7 xenograft tumor model, with the ERα protein decreasing by more than 90% by the end of the experiment. Moreover, more significant tumor growth inhibition (131% TGI) was observed when ARV-471 was combined with a cyclin dependent kinase 4/6 (CDK4/6) inhibitor, significantly decreasing ERα protein levels. In a patient-derived xenograft model with an *ESR1* Y537S mutation, ARV-471 completely inhibited growth at a dose of 10 mg/kg and significantly reduced the level of mutant ER protein, exhibiting superior inhibition compared with fulvestrant. In the future, ARV-471 will be studied as a monotherapy or in combination with other therapies, such as CDK 4/6 inhibitors.

#### 3.2.2 ERD-308

In 2019, Hu et al. (57) designed, synthesized, and evaluated a series of PROTAC degraders targeting ERα and successfully discovered a highly potent degrader, ERD-308 (**Table 1**), consisting of an ER antagonist and VHL-1 as an E3 ligase. DC_50_ values of ERD-308 in MCF-7 and T47D ER+ breast cancer cells were 0.17 and 0.43 nM, respectively, and more than 95% ERα degradation was induced at concentrations as low as 5 nM in both cell lines. Compared with fulvestrant or RAD1901, two conventional SERD molecules, ERD-308 achieved more thorough ERα degradation and more effectively inhibited cell proliferation in MCF-7 cells. Moreover, ERD-308 inhibited the expression of ER-regulated genes and the proliferation inhibition ability was much higher than that of fulvestrant and the SERM molecule raloxifene. The discovery of ERD-308 may promote the development of a completely new class of therapeutics to treat ER+ breast cancer.

#### 3.2.3 ERD-148

During the structure activity relationship (SAR) studies of the PROTAC degrader ERD-308, compound ERD-148 (**Table 1**) also displayed excellent degrading potency (57). ERD-148 has a hydrophobic linker, whereas ERD-308 has a polyethylene glycol unit (PEG) embedded in the linker. Further investigations were conducted to characterize the pharmacological activity of the PROTAC degrader ERD-148 in ER+, estrogen-dependent MCF-7 wild type, cY537S, and cD538G mutant cells (84). Results showed that ERD-148 inhibited the growth of ER-dependent cells, with IC_50_ values of 0.8 nM, 10.5 nM, and 6.1 nM, in MCF-7 wild type, cY537S, and cD538G mutant cells, respectively. ERD-148 significantly downregulated ERα expression at concentrations as low as 10 nM in MCF-7 wild type and Y537S mutant cells. Moreover, ERD-148 significantly downregulated the mRNA level of *Growth regulation by estrogen in breast cancer 1* (*GREB1*), an ER-regulated gene. However, ERD-148 did not inhibit the growth of ER-negative and estrogen-independent MDA-MB-231 breast cancer cells.

#### 3.2.4 Specific and Non-genetic IAP-Dependent Protein Eraser ERs

In 2011, Itoh et al. (93) designed specific small molecular protein degradation inducers known as specific and non-genetic IAP-dependent protein erasers (SNIPERs). SNIPER is a class of PROTAC used for protein degradation *via* the UPS. SNIPER comprises bestatin (BS), a ligand that interacts with the IAPs and an appropriate ligand for the target protein (94). Using 4-hydroxytamoxifen (4-OHT) as an ERα ligand, they developed a series of SNIPER(ER)s targeting the ERα protein for degradation, including SNIPER(ER)-3 (**Table 1**) (85, 86). SNIPER(ER)-3 potently induced the degradation of ERα and inhibited the estrogen-dependent expression of *presenili 2* (*pS2*) gene in ER+ estrogen-dependent MCF-7 cells. ER is degraded by proteasomes following cIAP1-mediated ubiquitylation. SNIPER(ER)-3 reduced the viability of MCF-7 cells expressing ERα, but not of U2OS and HeLa cells, which do not express ERα protein. SNIPER(ER)-3 also induced necrotic cell death, accompanied by the high mobility group box 1 (HMGB1) release, a necrosis marker, from the cells into the media. Unfortunately, BS is a nonspecific ligand that limits the bio-orthogonality and maximal potency of SNIPER(ER)s.

Several IAP antagonists were subsequently incorporated into SNIPER(ER)s, and a novel SNIPER against ER, called SNIPER(ER)-87 (**Table 1**), was developed (87). SNIPER(ER)-87 contains an LCL161 derivative as a ligand for IAP, reduces ERα protein levels at the nanomolar level, and preferentially recruits X-linked inhibitor of apoptosis protein (XIAP), but not cIAP, to ERα for degradation. SNIPER(ER)-87 induced more than 50% degradation of ERα at a concentration as low as 3 nM. Daily administration of SNIPER(ER)-87 suppressed tumor growth in the MCF-7 breast tumor xenograft model and induced ERα degradation in tumors. In addition, SNIPER(ER)-87 effectively inhibited β-estradiol-mediated ERα-dependent transcriptional activation.

#### 3.2.5 PROTAC-Like SERDs cpd. 17e

Li et al. (88) designed and synthesized a series of novel PROTAC-like SERDs containing an oxabicycloheptene sulfonamide (OBHSA) core structure and different side chains (the basic side chains, long alkyl acid side chains, and glycerol ether side chains). The basic side chain was confirmed as the appropriate degron, exhibiting the best anti-proliferative activity and good ERα degradation efficacies. Compound **17e** (cpd. 17e) (**Table 1**) was selected as the best compound. Notably, 1 μM of cpd. **17e** completely degraded ERα and 500 nM of the cpd. **17e** showed good degradation activity. Compared with the parent compound OBHSA-1, the basic side chain of cpd. **17e** played a pivotal role in increasing the potency of ERα degradation. These results suggest new possibilities for the development of more effective PROTACs.

### 3.3 Small-Molecule PROTACs Targeting Estrogen-Related Receptors

The estrogen-related receptor (ERR) is an orphan nuclear hormone receptor that can produce biological functions without binding to ligands. There are three ERR subtypes: ERRα (NR3B1), ERRβ (NR3B2), and ERRγ (NR3B3). Recent studies (95, 96) have found that ERRα is closely associated with estrogen-induced breast cancer, endometrial cancer, and other estrogen-dependent tumors. Both *in vitro* and *in vivo*, pharmacological inhibition and gene knockout of *ERRα* have been shown to slow down the progression of breast cancer (97, 98).

#### 3.3.1 PROTAC cpd. 29

To design the effective small molecule PROTAC, Bondeson et al. (43) replaced the hypoxia-inducible factor 1α (HIF1α) peptide with a high-affinity small-molecule ligand of VHL, retaining the essential hydroxyproline part for VHL binding. A thiazolidinedione-based ligand, selectively binding to ERRα over other reported ERR isoforms, was incorporated to generate PROTAC cpd. **29** (**Table 1**). PROTAC cpd. **29** decreased the level of ERRα in MCF-7 breast cancer cells in a dose-dependent manner. The D_max_ was 86%, and the DC_50_ was approximately 100 nM. *In vivo* experiments showed that PROTAC cpd. **29** possessed broad tissue distribution and knocked down ERRα in tumor xenografts. These results show that PROTACs offer a method for achieving *in vivo* protein knockdown with potential therapeutic applications.

#### 3.3.2 PROTAC cpd. 6c

Peng et al. (89) designed and synthesized a series of (E)-3-(4-((2,4-bis(trifluoromethyl)benzyl)oxy)-3-methoxyphenyl)-2-cyanoacrylamide derivatives as new ERRα degraders based on the PROTAC concept, and cpd. **6c** (**Table 1**) was identified as one of the most potent and selective ERRα degraders. Cpd. **6c** induced remarkable degradation of ERRα at a concentration of 3.0 nM. Additionally, 30 nM of cpd. **6c** specifically degraded > 80% of ERRα and potently decreased the levels of proteins encoded by ERRα downstream target genes. Additional studies suggested that ternary complex and ubiquitin-proteasome were involved in cpd. **6c**-mediated ERRα degradation.

### 3.4 Small-Molecule PROTACs Targeting Other Proteins in Breast and Prostate Cancers

#### 3.4.1 ARV-771

Inhibitors of BET proteins have recently shown growth-inhibitory activity in preclinical models of CRPC, with BET being an attractive target in CRPC (99). In 2016, ARV-771 (**Table 1**), a VHL E3 ligase-based pan-BET PROTAC, was designed and synthesized (90). It rapidly degraded bromodomain protein 4 (BRD4) protein, with DC_50_ < 5 nM, and inhibited the expression of c-Myc, with IC_50_ < 1 nM in 22Rv1, LnCaP95, and VCaP CRPC cell lines. However, the ARV-771 downregulating activity of c-Myc expression was more than ten times higher than that of the BET inhibitor, JQ1. Moreover, ARV-771 induced significant poly (ADP-ribose) polymerase (PARP) cleavage, caspase activation, and apoptosis in 22Rv1 cells. Additionally, ARV-771 was efficacious in two different xenograft models of CRPC and resulted in tumor regression in enzalutamide-resistant 22Rv1 tumor xenografts.

#### 3.4.2 PROTAC cpd. 3

Zhao et al. (91) designed and synthesized a small PROTAC molecule, the representative cpd. **3** (**Table 1**), based on niraparib as the PARP1 binding moiety and nutlin-3 derivatives as the E3 ligase-binding moiety. PROTAC cpd. **3** induced PARP1 cleavage and apoptosis in the MDA-MB-231 cell line. PROTAC cpd. **3** was also 5-fold more potent than niraparib, olaparib, and veliparib at degrading PARP1 when tested in MDA-MB-231 cells and exhibited no cytotoxicity to normal breast cells. This PARP1-targeting PROTAC-type compound represents a huge potential application value in the therapy of the MDA-MB-231 cell-like subtype of triple-negative breast cancers.

## 4 Discussion

Steroid hormones play vital roles in the initiation and progression of sex hormone-dependent cancers, including breast and prostate cancers. Endocrine therapy targeting ERs, ARs, and ERRs represents a potential and pivotal therapeutic strategy for breast/prostate cancer therapy. The PROTAC approach is a novel therapeutic strategy that is particularly suited for abolishing the activity of target proteins, including ERs, ERRs, ARs, as well as other oncoproteins. Generally, PROTACs bind only to a small proportion of their target proteins and act like a catalyst, which is highly effective at degradation even if the target protein’s concentration varies considerably within cells. However, traditional small molecule regulators play their roles by occupying the active pocket sites and require high drug administration dosage to maintain activity, which increases the risk of off-target and adverse effects. Protein degradation using PROTACs also provides the opportunity to overcome resistance to endocrine therapy and receptor antagonists in sex hormone-dependent cancers. To improve cell permeability and stability, PROTACs have evolved from peptides to small molecules. Many successful cases of small molecule-based PROTACs have recently been reported in cultured cells, mice, and humans, demonstrating the feasibility of applying PROTACs in clinical settings.

### 4.1 Challenges and Limitations

Although PROTACs offer considerable advantages and promising prospects in the clinical treatment of prostate and breast cancers, particularly hormone-resistant prostate and breast cancers, some issues still require consideration. First, PROTACs have a relatively high molecular weight (typically 700–1100 Da) and may not conform to Lipinski’s ‘rule of five’, which is a rule of thumb for evaluating whether a small molecule possesses pharmacological or biological properties of an orally active drug in humans (100). The high molecular weight of PROTACs reduces their cell permeability, tissue penetration, and metabolic attack, posing challenges for oral administration. To overcome the high molecular weight nature of typical PROTACs, heterobifunctional PROTACs can be formed intracellularly through the bio-orthogonal click combination of two tagged small molecule precursors in cells and can successfully induce target protein degradation, named in-cell click-formed proteolysis-targeting chimeras (CLIPTACs) (101).

In addition, it is cumbersome to predict the degradation efficiency of small-molecule PROTACs. For target protein ligands, high binary binding affinities do not always yield efficient degradation of target proteins (40), suggesting that it is difficult to identify the best ligand of the target protein for PROTAC construction. Linkerology plays the most pivotal role in PROTAC components, determining the biological and physicochemical properties of PROTACs. Optimization of length and rigidity of linker units are important for improving pharmaceutical performance. It is difficult to select an optimal linker tethering site on ligands of the target protein, even when the co-crystal structures of a specific ligand and the target protein are available.

Unintended on-target and off-target toxicities can also affect the clinical translation of PROTACs (102). E3 ligases contribute to the specificity in the degradation of target proteins. Complete degradation of certain proteins and degradation of untargeted proteins in a complex or close proximity to E3 ligase may be detrimental. The unintended on-target and off-target toxicities also affect PROTACs’ clinical translation (102). These toxicities can be avoided by incorporating a suitable E3 ligase, which is tissue-specific and tumor-selective, in the design of selective PROTACs. Light-controllable photo-PROTACs, whose action can be controlled under an external source of light to direct tumor-specific degradation of target proteins, have been reported by several research groups. Photo-PROTACs increase the potential for target degradation in the desired tissues.

The “hook effect” is another unavoidable problem. At high intracellular concentrations of small-molecule PROTACs, binary complexes are favored over ternary complexes, ultimately decreasing target degradation (16, 103). Since some target protein ligands have agonist/antagonist activities, PROTACs can function as traditional small molecule activators/inhibitors. This effect has been observed where PROTAC targets AR-containing mutations within the ligand-binding domain, as AR antagonists act as agonists for these mutations (104, 105). Consequently, generating tumor-specific/selective PROTACs is essential for reducing on- and off-target toxicities, while identifying the tumor-specific/selective E3 ligase is of primary importance. Thus, it is advisable to use suitable PK-PD models to predict the PROTAC dosage to avoid the hook effect.

### 4.2 Prospects

Twenty years after the first PROTACs were synthesized in 2001, small-molecule PROTACs have not only drawn the attention of academic researchers, but also of pharmaceutical companies. Although there are concerns over their relatively high molecular weights and low oral availability, two orally active small-molecule PROTACs, ARV-110 (an AR degrader) and ARV-471 (an ER degrader), have been studied in phase I/II clinical trials. Small-molecule PROTACs are a potential strategy for drug discovery, providing a new way to treat sex hormone-dependent cancers. Since immune checkpoint inhibitors have achieved great success against some tumors by enhancing the antitumor immunity of immune cells, researchers inspired by this have attempted to design PROTACs that can potentially strengthen antitumor immunity (106, 107). Future development of small-molecule PROTACs shall focus on more “undruggable” proteins, and new E3 ligases with tissue-specific and tumor-specific expression patterns may be recruited. Overall, it is foreseeable that PROTACs will soon benefit patients.

## Author Contributions

YZ, HX, and YW designed the concept of the review and participated in manuscript writing, editing, revising, prepared the figures and supervised the entire process. LS, ZW, and LL collect literature data, participated in manuscript writing and formatting. LL and YZ prepared the structure of compounds. LL and JZ participated in manuscript writing, proof editing, and graphical design. All authors contributed to the article and approved the submitted version.

## Funding

This work was supported by Excellent Youth Fund of Sichuan Cancer Hospital [grant number YB2021024], and Science and Technology Program of Sichuan Province [grant number 2021YFH0145], Scientific Research Project (Foundation) of the Health Planning Committee of Sichuan [grant number 20PJ108], and Scientific research project of Sichuan Medical Association [grant number S20023].

## Conflict of Interest

The authors declare that the research was conducted in the absence of any commercial or financial relationships that could be construed as a potential conflict of interest.

## Publisher’s Note

All claims expressed in this article are solely those of the authors and do not necessarily represent those of their affiliated organizations, or those of the publisher, the editors and the reviewers. Any product that may be evaluated in this article, or claim that may be made by its manufacturer, is not guaranteed or endorsed by the publisher.
